# Fostering interdisciplinary working within global surgery at an undergraduate level: A hackathon based approach

**DOI:** 10.1016/j.sopen.2023.07.021

**Published:** 2023-08-09

**Authors:** Vigneshwar R. Veerappan, Niraj S. Kumar, Jashan Selvakumar, Mehak Kakwani, Katya M.A. Marks

**Affiliations:** a Hull York Medical School, University of York, UK; b National Medical Research Association, London, UK; c St George's, University of London, UK; d Leeds School of Medicine, University of Leeds, UK; e Johns Hopkins Bloomberg School of Public Health, United States of America; f University of Oxford Medical School, UK

**Keywords:** Global surgery, Surgical education

## Abstract

**Objective:**

To investigate the effectiveness of a virtual hackathon in fostering interdisciplinary working amongst undergraduate students in global surgery.

**Methodology:**

In this study, we developed a 3 day event consisting of guest lectures, a documentary screening and a hackathon supported by academics and experts in the field, to provide students with the opportunity to learn more about and work in interdisciplinary teams within global surgery. Students had the option to attend just the lectures or both the lectures and hackathon. Quantitative and qualitative results were collected through a pre and post session survey.

**Results:**

A total of 21 responses were received for the hackathon and 26 responses for the general event (response rate for event = 26 %, response rate for hackathon = 24.7 %). There was a significant improvement in understanding of interdisciplinary working in global surgery between the pre and post-session survey, with an increase in median from 3 (IQR = 2–3.5, n = 21) to 4 (IQR = 4–5, n = 21) (p < 0.05). Respondents noted that the benefits of a hackathon were that it was very engaging, and brought in diversity of thought and expertise. The drawbacks to the hackathon were that it was fast-paced, required prior knowledge and the virtual platform it was hosted on.

**Conclusion:**

Our study demonstrates that hackathons are an effective, inclusive and equitable way for students to engage in and learn about interdisciplinary working. It is important that as institutions recognise and develop global surgery courses, these courses reflect the interdisciplinary nature of the field.

## Introduction

In recent years, the need for improved surgical provision in low- and middle-income countries (LMICs) has reached the forefront of discourse surrounding equitable healthcare provision and reduction of treatable morbidity worldwide. This is in line with a movement towards ‘global surgery’, whereby multidisciplinary stakeholders aim to provide improved and accessible surgical care globally utilising and enhancing international healthcare provision systems, requiring a close overlap between the three core areas of outreach, advocacy, and research [1].

Despite limited exposure to global surgery via clinical rotations in LMICs and few formalised educational opportunities available within the field, medical students and trainee doctors have proven keen to pursue global surgery and explore opportunities, such as those created by student global surgery societies. A previous report showed a global surgery course developed in North America to provide formal graduate education aimed at providing interdisciplinary teaching based on the focus of the Lancet commission. Following suit, other North American and European global surgery programs are being offered to highlight the career to prospective global surgeons [2].

However, for students interested in the field there has been little effort undertaken to provide didactic opportunities between peers, crucial to ensure that they are prepared to understand the diverse challenges in the field and allow greater interaction amongst a population of learners and educators from varied backgrounds, regardless of whether they are able to participate in these formalised global surgery teaching offerings. This limited exposure to global surgery means that there is cause for concern as to how the future generation of aspiring surgeons will be prepared to understand and tackle the challenges faced in global surgery.

To mitigate this, we sought to develop a hackathon aimed to explore an interdisciplinary understanding of current priorities and how best we can use current resources to develop capacity and improve outcomes. Hackathons are events whereby individuals from different background are brought together and work on solutions to different problems [3]. These solutions are then pitched to a panel of judges. Although historically within fields of computer science and engineering, this model has been tested and demonstrated to facilitate the creation of solutions based on combined experiences from disciplines and has shown value when used in the context of global surgery in a developing country [3].

In this study we describe the development and evaluation of an interdisciplinary global surgery hackathon aimed for medical students and trainees to develop an understanding of problems and priorities currently being targeted. We aim to describe the impact of such a hackathon on student knowledge of interdisciplinary global surgery and how it may be used to improve surgical provision and raise greater awareness for global surgery amongst the next generation of global surgery practitioners.

## Methods

### Event format

The event hosted by Lifebox Global Surgery Alliance (Lifebox GSA) and InciSioN UK, was held virtually over the weekend of March 26th–28th 2021. The event welcomed students and professionals, aiming to attract individuals from a variety of disciplines relevant to global surgery. The event consisted of a screening of *The Checklist Effect*, six keynote speakers sessions and a hackathon. Conference keynote speakers were invited from a variety of disciplines: politics and policy making, ethics and law, academia and surgery, epidemiology and statistics, engineering, computer science and innovation, advocacy and NGO. Attendees had the option to attend the lectures and hackathon or only lectures.

The documentary screening and lectures were delivered using zoom webinar application, and the hackathon session was facilitated through zoom breakout rooms. The Hackathon Networking Session was hosted by MedTech Foundation and held on Friday 26th March to open the conference. A program of enrichment talks were delivered by MTF and GASOC to Hackathon participants, on Concept Generation, Frugal Innovation and the GASOC organisation. Hackathon participants were separated into interdisciplinary teams, with each team assigned one of three unmet needs.

The unmet needs were set as follows:

1) ‘What strategies can improve Uptake and Fidelity of Safe Surgery Checklist Use?’ - Set by representatives from Ariadne Labs and the University of Calgary's EQuiS Research Platform
2) ‘What would you do to bridge the education and awareness gap in precision medicine?’ - Set by representatives from McCann Health
3) ‘What would you do to help achieve better patient follow up for patients who have returned to homes far away?’ - Set by a representative from Stanford University and ReSurg International.

Each hackathon team was provided also with a google drive folder containing orientation material: video explanation of their unmet need, Hacking Global Surgery program, Hacking Global Surgery mentor list, MTF ‘Hackathon Participant Guidance’ document, MTF Concept Note template, MTF ‘A Brief Introduction to Slack’ document, video introduction to their assigned unmet need.

Teams were provided with a total of 4 h and 40 min of dedicated innovation time, with the opportunity to continue work between sessions. Innovation sessions were guided by mentors from Ariadne Labs, EQuiS research, InciSioN UK, Lifebox GSA, GASOC and MTF, who could drop in on team discussions.

On the last day, teams were given 5 min to pitch their proposal to an audience of their fellow conference delegates and three judges. The hackathon was judged by representatives from Stanford University and ReSurg, Envisionit Deep AI, and Hacking Health. Judging was conducted based on three criteria: clarity of the pitch, feasibility of the idea, potential impact of the idea.

The entire event was free to attend, with a voluntary option to donate to the Lifebox Foundation. Sessions were scheduled, wherever logistically possible, to be inclusive of varied time zones.

### Event promotion

Given that this event was aimed at fostering interdisciplinary working amongst university students, the event promotion strategy was devised to have as wide a coverage as possible and attract students from a range of undergraduate courses. Social media strategy involved weekly event promotion through the twitter and Facebook pages of Lifebox GSA and InciSioN UK. In addition to this, the conference team discussed a list of relevant student societies and undergraduate courses for whom this event may be relevant. UK university student societies such as medical societies, engineering societies, law-based societies etc. were contacted by our team to advertise and promote the event. Both InciSioN UK and Lifebox GSA's student representative network was also utilised to deliver targeted event promotion to university students.

### Data collection

Pre and post session surveys were disseminated to all hackathon attendees, aimed at evaluating the utility of the event in improving understanding of interdisciplinary working in global surgery. One pair of surveys addressed the general event (keynote sessions and documentary screening) and one pair addressed the hackathon specifically. Hence, those who attended the hackathon received 2 separate pre and post session surveys, one for the general event (keynote lectures and documentary screening) and one for the hackathon specifically. Answers were collected using a Likert scale (5-very helpful/very good understanding, 1-not helpful at all/no understanding at all).

### Data analysis

The responses collected to the questions were quantitatively analysed and a paired *t*-test was used to compare respondents' pre and post session survey responses. An independent t-test was also used to compare responses on understanding of interdisciplinary working in global surgery between respondents who attended the lectures only and those who attended lectures and hackathon.

Qualitative data was collected from the feedback form using the 3 following questions:

1. How can interdisciplinary working be fostered more within the field of Global Surgery in academic and professional institutions?
2. In your experience, what were some of the benefits of learning about Global Surgery through a Hackathon styled event?
3. In your experience, what are the disadvantages of learning about Global Surgery through a Hackathon styled event?

Qualitative analysis used Braun and Clarke's reflexive thematic analysis approach. Two authors familiarised themselves with the data and independently generated initial codes through an inductive process. The data was initially divided into ‘benefits’ and ‘areas of improvement’. Any differences in interpretation were discussed and agreed by consensus. Participant data were interpreted and summarised. Codes of similar information were merged leading to a series of phenomena that appeared increasingly representative of the participants' perspectives.

### Conflict of interest

The event had speakers, judges and hackathon facilitators from a wide array of backgrounds, organisations and industries, to ensure the event was delivered in a truly interdisciplinary fashion.

However, the team involved in the data collection, analysis and write up of this paper were students and members of InciSioN UK or Lifebox GSA, and were not affiliated with any organisation or industry involved in the design, delivery and facilitation of the hackathon and associated lectures. This has enabled the team involved in the write up of this paper to mitigate ensure there are no conflicts of interest.

## Results

### Quantitative results

A total of 100 and 85 participants registered to attend the event and hackathon respectively. Participants included medical students and allied healthcare workers from across the world. A formal survey on participant demographic was not undertaken. Pre and post session surveys were sent to all participants.

### Quantitative analysis

**Fig. 1:**
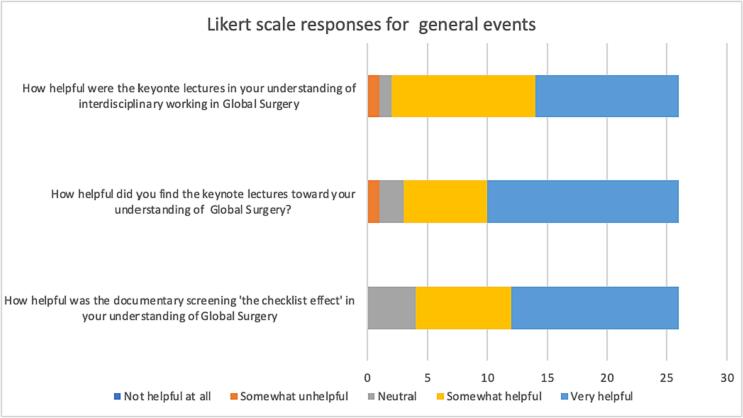
Likert scale responses for general events.

A total of 21 responses were received for the hackathon and 26 responses for the general event (response rate for event = 26 %, response rate for hackathon = 24.7 %). Participants who received the post-session survey on the general event were asked to rate how helpful they found the sessions other than the hackathon (documentary screening and keynote lectures) to their understanding of global surgery and interdisciplinary working in global surgery (Fig. 1).

A majority of participants (n = 22) found the documentary screening somewhat or very helpful to their understanding of global surgery with a median of 5 (IQR = 4–5, n = 26). Majority of participants also found the keynote lectures somewhat helpful or very helpful to their understanding of global surgery and interdisciplinary working in global surgery with a median of 5 (IQR = 4–5, n = 26) and 4 (IQR = 4–5, n = 26).

**Fig. 2:**
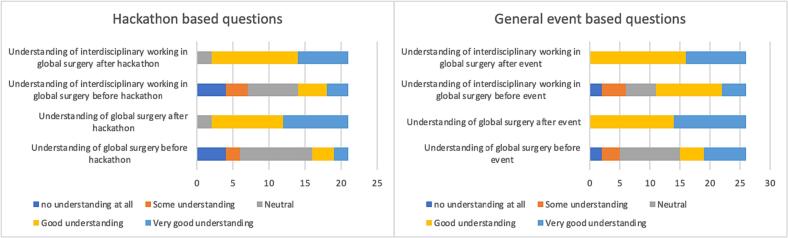
Likert scale responses for hackathon based questions and general event based questions.

Participants were asked to rate their understanding of global surgery and interdisciplinary working within global surgery before and after the session. These questions were asked in surveys that were sent out for the hackathon and the general event (Fig. 2).

Pre-session survey of participants who attended the hackathon showed wide variation in response regarding understanding of interdisciplinary working in global surgery, with a median of 3 (IQR = 2–3.5, n = 21), whereas post session surveys showed that a majority of participants had a good or very good understanding of global surgery and interdisciplinary working in global surgery with both having a median of 4 (IQR = 4–5, n = 21). A paired *t*-test demonstrated significantly higher understanding of global surgery and interdisciplinary working in global surgery in participants after the hackathon as compared to before (p < 0.05).

The trend was similar in the pre and post session surveys regarding the general events, with a median of 3 (IQR = 3–5, n = 21) when responding to understanding of interdisciplinary working within global surgery prior to the event, and a median of 4 (IQR = 4–5, n = 21) for the same question in the post-session survey. A paired *t*-test applied to the pre and post session surveys of participants in the general events survey also showed a significantly higher understanding of global surgery and interdisciplinary working in global surgery after the general event as compared to before (p < 0.05).

An independent *t*-test showed no significant difference in participant understanding of global surgery and interdisciplinary working in global surgery in the post-session survey when compared between those who responded to the hackathon and those who responded to the general event.

### Qualitative analysis

#### Benefits

##### Engaging

The Hackathon, where individuals were encouraged to create a feasible solution to a real-life global health problem, proved to be a challenging, yet intellectually stimulating task for the attendees. Participants reflected on it being a steep learning curve and during the process, becoming aware of a lot of topics they were previously uninformed about (Table 1).

**Table 1:** Themes identified based on free text responses.

Themes	Sub-themes	Quotes
Benefits	High level of engagement	“*It was very useful to discuss the global surgery issues with people who had different levels of experience, and experience beyond medical school.*”
	Diversity in thought	“*It fosters that sense of partnership and equity needed in tackling global surgery challenges in a healthy way.*”
	Diversity in expertise	“*Projects on the curriculum linking students from medical schools to students doing different degrees will encourage collaboration with other disciplines*”
Drawbacks	Lack of prior knowledge	“*At some points, it felt like because of our own massive lack of knowledge, we were just guessing and assuming for a a large part of the project.*”
	Fast-paced	“*Very hands on, this was good. Very fast, which meant it was hard to take in everything and quickly start spit-balling ideas to make something; just because the timing was strained (a bit)*”
	Virtual platform	“*(There were) challenges to doing this online*”

This active learning style, several participants expressed, helped them remember the key messages taught during the sessions and consider how they may be implemented in a practical manner, rather than just being considered in theory. Some went on to mention that having such events more often would be beneficial to nurture interdisciplinary collaboration within their own team at work and internationally.

“These collaborations can effectively answer global surgery questions”

“Further collaborations to foster the sustainable development goals towards the future of health.”

##### Diversity in thought

Delegates across the world were in attendance at the global health events and hackathon, which made the Hackathon experience, in particular, far more enriching for everyone involved. Active discussions throughout the weekend helped participants understand and reflect on different standpoints about certain pressing issues, as well as provide their own perspectives to help figure out solutions.

“It helps one see problems and solutions through different lens.”

The team spirit created with the competition element of the Hackathon, with teams striving to discover the most practical solution to the global health issue, helped initiate teamwork and collaboration. These soft-skills, delegates reflected, would be beneficial to their wider clinical teams as it helped them recognise the importance of teamwork and optimising the expertise of team members.

Participants recognised that the sessions and Hackathon, not only, highlighted the successes in global health and interdisciplinary working but the challenges in the process; both important for moving forward and making change.

##### Diversity in expertise

The majority of attendees cited interdisciplinary collaboration as one of the primary benefits for participating in such an event. The benefits (also stated above) being: having different views, levels of expertise and resources at their disposal.

A few delegates reflected further on the benefits of working alongside other healthcare professionals and academics involved in the field of global surgery, by mentioning the importance of integrating this type of learning and events within the educational curriculum. Establishing a starting point for teaching healthcare professionals and academics about global health through an introductory module/curriculum on global health could further enhance interdisciplinary, international collaboration.

#### Areas for improvement

##### Lack of prior knowledge

The most prominent issue experienced by several attendees was the lack of knowledge they had prior to the hackathon, which meant they were sometimes dependent on other individuals in the groups. Although this could foster better teamwork, it relies on at least one individual having the expertise on the particular hackathon subject to have optimal engagement.

##### Fast-paced

Stemming from this, some delegates felt that there was not enough time to grasp the complex concepts that were introduced in the hackathon. This was particularly experienced by those who have no prior awareness of global surgery and what it is. These individuals felt that it was more challenging for them to come up with solutions and make decisions. Together, this made the experience more “intense” and “time constrained” and at times, “daunting” because they felt as if they were lagging behind their group.

##### Virtual platform

Having a virtual online hackathon made it more globally inclusive and efficient but a few delegates expressed that there were challenges with this format when working in a team. One issue was a few groups had a few attendees not turning up for the entirety of the hackathon which meant it put more workload on the other participants. Furthermore, some felt as though they found it harder to develop meaningful interpersonal connections with their team, due to the platform being online, which made it difficult for them to cultivate these relationships.

## Discussion

The results demonstrate that every component of this event; hackathon, lectures and documentary screening significantly improved attendees' understanding of global surgery and interdisciplinary working within global surgery. All components of the hackathon were equally good at developing understanding of global surgery and interdisciplinary working through global surgery. Respondents felt that the benefits of a virtual hackathon were that it is very engaging, required an interdisciplinary approach and promoted a more inclusive and collaborative approach to global surgery. However, some of the disadvantages was that the hackathon was fast paced, required some prior knowledge, and the virtual platform posed a barrier for participants to develop more interpersonal connection.

This event was organised to provide a platform for students and early career professionals to gain an understanding of interdisciplinary working within global surgery. While global surgery has only been in the forefront for the past few years since the Lancet commission on global surgery, there have been several studies that have highlighted the importance of interdisciplinary and collaborative working within global surgery [4,5]. Dare et al., in a 2014 article, discussed how the delivery of adequate surgical care is not only reliant on surgeons and physicians, but also requires other facets of healthcare such as access to markets, transport, power and political and economic stability to work in tandem [6]. Even within the operating theatre, frugal innovations such as low-cost laparoscopes or gasless laparoscopic procedures require surgeons to work in an interdisciplinary fashion with other professionals such as engineers and technologists. Given the lack of global surgery education within universities and surgical training programs, students and early career professionals not only lack knowledge about global surgery as a field, but also lack opportunities to collaborate with professionals outside of surgery and medicine to achieve [[7], [8], [9]].

Several studies have explored avenues to introduce interdisciplinary working within medical or surgical training, one of these avenues being hackathons [2,10,11]. In a study by Silver et al. involving a hackathon for rehabilitative healthcare, the authors noted how the ability of hackathons to bring professionals from different industries together will have long lasting implications as it will allow physicians to navigate healthcare problems in an interdisciplinary way in their own practice after the hackathon [12,13]. When applying hackathons to surgical care, Mithra et al. noted that hackathons enable the development of an innovative ecosystem with a culture for interdisciplinary working and innovation [11]. Hackathons not only bring in diversity of expertise, but also a diversity in participants in terms of gender, ethnicity and geography, enabling participants to approach problems in an innovative and equitable way [13]. Our study largely corroborated the findings that hackathons are useful in fostering innovative and interdisciplinary approaches within participants. However, it is also important to recognise the limitations to organising a hackathon. A truly interdisciplinary hackathon requires a significant amount of time and resources to organise, as well as an advertising strategy that attracts participants from various different professional backgrounds. This can be challenging to implement, especially in a low resource setting. As our study found, participants identified their lack of prior knowledge on global surgery to be a barrier to participating meaningfully in the hackathon. A hackathon without adequate interdisciplinary representation risks participants not being able to acquire the necessary skills or knowledge, or developing solutions that are not feasible within a given setting.

Besides hackathons, Fitzgerald et al. also demonstrated the effectiveness of combining didactic guest lectures with hands-on activities such as developing a National Surgical and Obstetrics Plan (NSOAP) for a country of their choice, fostering an interdisciplinary approach amongst students [2]. Similarly, an interdisciplinary approach can be fostered by developing research projects with multiple senior or supervising authors from different professional backgrounds, allowing early career researchers to work with experts from different fields, gaining the knowledge and skills around interdisciplinary working, although this will require an active effort on part of senior researchers [14]. While our study found hackathons to be helpful in participant's understanding of interdisciplinary working, we found no significant difference in understanding between participants who attended the hackathon and lectures and participants who attended only the lectures, corroborating findings that lectures can be an equally viable way of fostering interdisciplinary working.

However, there are some limitations to our study. Firstly, our study has a small number of responses and data on participant background was not analysed. Larger cohort research is required to further understand the impact of hackathons on knowledge of interdisciplinary working. This can be organised through interdepartmental collaborations within universities and integrated within the educational courses organised at universities. Secondly, we were unable to longitudinally evaluate the impact of the hackathon or an improved understanding of interdisciplinary working on the participants' professional practice. Finally, as this initiative was organised by largely medical student led groups, the article is written from the perspective of medical professionals. As such, there were barriers to recruiting participants from other backgrounds. While our event promotion strategies took this into account and made deliberate attempts to expand the reach of promotion, underrepresentation of participants from certain professions may have persisted.

### Future direction

This event has added to the growing body of literature demonstrating innovative ways to foster interdisciplinary working within global surgery, but importantly provides a perspective of how this can be done at an undergraduate level. Given the number of young people working in the field of global surgery, the need to engage in interdisciplinary working at an undergraduate level is more pertinent [15,16]. Given how effective the hackathon model is at providing insight into interdisciplinary working within global surgery, this event can be organised at a larger scale with more participants. Running this event in person may also prove more effective as it will mitigate some drawbacks identified here. Where organising hackathons are not feasible, our study has shown that interdisciplinary lectures can be equally effective. Hence, universities or organisations within global surgery can organise simple interdisciplinary lectures, such as having a lecture on frugal engineering, or health policy within global surgery delivered to an audience of medical students.

## Conclusion

As work towards delivering surgical care around the world increases, there is a need to improve education on global surgery at an undergraduate level. It is important to recognise that delivering surgical care requires the concerted efforts of various different professions, industries and stakeholders outside physicians and surgeons. While this has been recognised, there is still a notable lack of education on interdisciplinary working in global surgery at an undergraduate level. Our study demonstrates that hackathons are an effective, inclusive and equitable way for students to engage in and learn about interdisciplinary working. It is important that as institutions recognise and develop global surgery courses, these courses reflect the interdisciplinary nature of the field.

## Ethical approval statement

Ethical approval was not required as data was collected as part of anonymous feedback for the 3-day hackathon.

## Funding

This research has received no specific grant from any funding agency in the public, commercial, or not-for-profit sectors.

## CRediT authorship contribution statement

VV and KM were responsible for the conception of this project. All authors contributed equally to all parts of the data collection, analysis and manuscript write up.

## Declaration of competing interest

The authors have no competing interests.
